# One-shot learning for autonomous aerial manipulation

**DOI:** 10.3389/frobt.2022.960571

**Published:** 2022-10-05

**Authors:** Claudio Zito, Eliseo Ferrante

**Affiliations:** ^1^ Autonomous Robotics Research Centre, Technology Innovation Institute, Masdar City, United Arab Emirates; ^2^ Department of Computer Science, Vrije Universiteit Amsterdam, Amsterdam, Netherlands

**Keywords:** robotics, one-shot learning, aerial manipulation, single and collaborative transportation, aerial grasping

## Abstract

This paper is concerned with learning transferable contact models for aerial manipulation tasks. We investigate a contact-based approach for enabling unmanned aerial vehicles with cable-suspended passive grippers to compute the attach points on novel payloads for aerial transportation. This is the first time that the problem of autonomously generating contact points for such tasks has been investigated. Our approach builds on the underpinning idea that we can learn a probability density of contacts over objects’ surfaces from a single demonstration. We enhance this formulation for encoding aerial transportation tasks while maintaining the one-shot learning paradigm without handcrafting task-dependent features or employing ad-hoc heuristics; the only prior is extrapolated directly from a single demonstration. Our models only rely on the geometrical properties of the payloads computed from a point cloud, and they are robust to partial views. The effectiveness of our approach is evaluated in simulation, in which one or three quadcopters are requested to transport previously unseen payloads along a desired trajectory. The contact points and the quadcopters configurations are computed on-the-fly for each test by our approach and compared with a baseline method, a modified grasp learning algorithm from the literature. Empirical experiments show that the contacts generated by our approach yield a better controllability of the payload for a transportation task. We conclude this paper with a discussion on the strengths and limitations of the presented idea, and our suggested future research directions.

## 1 Introduction

There is evidence that humans possess an internal model of physical interactions that enables us to grasp, lift, pull, or push objects of diverse nature in various contexts (Mehta and Schaal, 2002). It is also evident that such internal models are constructed over time as an accumulation of experience as opposed to an inherent comprehension of physics. In this paper, we investigate an internal model for enabling autonomous quadrotors equipped with cable-suspended passive grippers to generate contacts with unknown payloads for aerial transportation. By unknown payloads, we mean that our approach does not require a full CAD model of the object or information relative to its physical properties, such as its centre of mass (CoM) or friction coefficients. We only rely on geometric features extrapolated from vision. As the human’s internal model, our solution is not failure-free but provides a way to generate candidate grasps even when no information is available.

The hype for aerial manipulation has reached a high-fever pitch. Unmanned aerial vehicles (UAVs), such as quadrotors, have been recently at the centre of attention of the scientific community as the next means of autonomy. Their dynamic simplicity, manoeuvrability and high performance make them ideal for many applications ranging from surveillance to emergency response. More recently, such systems have been investigated for aerial transportation of payloads by towed cables. Small-size single and multiple quadrotors have been employed for load transportation and deployment by designing control laws for minimum swing and oscillation of the payload (Sreenath et al., 2013; Li et al., 2021a; Li et al., 2021b). Although in their dawn, current approaches disregard the generation of the contact points. Single drones assume point-mass loads to simplify the effects of the dynamics, and multiple drones are manually attached nearby the vertices or edges of the load, following the intuition of maximising the moments exercised on the object. No aerial system is capable of generating on-the-fly contacts, and it is not clear how the proposed controllers can cope with different payloads or contact configurations.

On the other hand, we have witnessed a growing interest in robot grasping and manipulation tasks in the last decade (Bohg et al., 2014; Stüber et al., 2020). Although we are still far from robots freely manipulating arbitrary objects, several promising solutions have been proposed over the years (Brahmbhatt et al., 2019; Sun et al., 2022). Google employed a dozen robots interacting in parallel with their own environment to learn how to predict what happens when they move objects around (Levine et al., 2018). However, collecting such a large amount of data for any task is very hard, and many researchers have focussed on more practical solutions. Additionally, the task of aerial manipulation presents another significant challenge–failed attempts may irreparably damage the payload and the AUVs. A more appealing approach is to learn models in a one-shot or a few-shot fashion when possible. Such approaches typically employ generative models which learn probability densities from demonstrations. We substantially reduce the searching effort when facing novel contexts by providing one or a few examples of a good solution. Furthermore, when the learning space is constructed over local features, the models tend to have a good generalisation capability within and across object categories (Kopicki et al., 2016).

In (Kopicki et al., 2016; Arruda et al., 2019), the authors formulate dexterous grasps for a humanoid robot as contacts between the robot’s manipulative links and the object’s surface. Only local surface properties of the object’s geometrics are required to learn the model, such as curvatures on the (local) contact patches. The model learns grasp types but does not encode task-dependent features or physical properties of the objects, and it has only been demonstrated for pick-and-place tasks. For different tasks beyond pick-and-place, the authors enable the possibility of encoding handcrafted loss functions, which requires (a rarely available) insight knowledge by the user on the specific task. In (Stüber et al., 2018; Howard and Zito, 2021), this approach has been extended to enable a robot to make predictions over push operations for novel objects. One-shot learning is utilised for identifying the contacts between the pusher and the object, and between the object and the environment, but an extra *motion model* needs to be learned from real or simulated experience in order to make the predictions. In practice, the motion models encode the task. Given the initial contact models, motion models are designed as conditional probabilities, following the general idea that making predictions on familiar initial conditions will yield more robust solutions. A few dozens examples are sufficient to learn a motion model for planar push operations since the environment constrains the motion. Such luxury is not available for aerial manipulation, and it would require us to approximate the drone-payload system dynamics to make any prediction.

**FIGURE 1:**
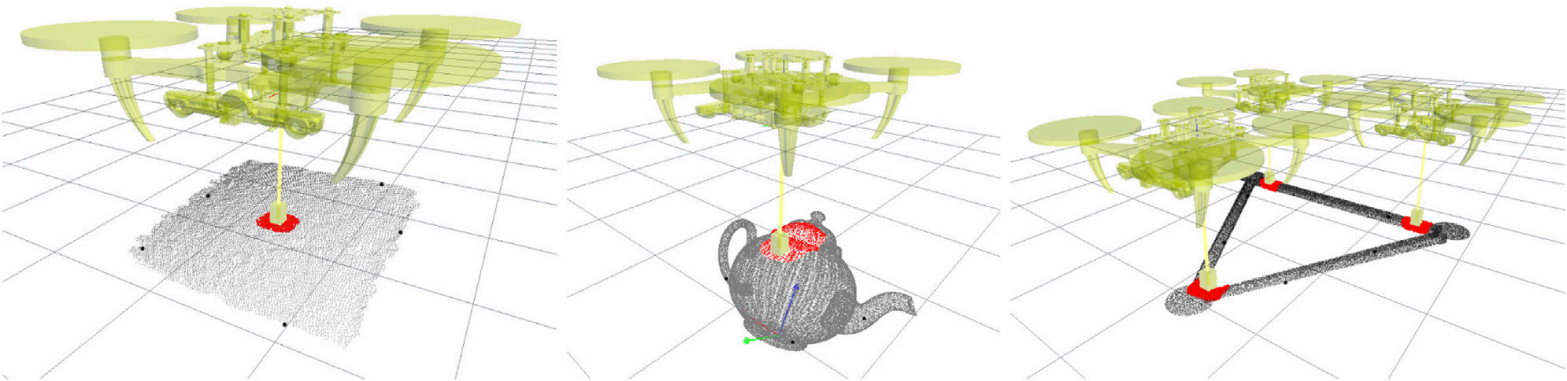
Learned contact models. (left) shows a central contact with a flat surface, i.e. top face of the Fedex box, (middle) shows a curved contact on a teapot, and (right) a triangular configuration for three drones. The red points represent the sub-sampled local area of the surface used to learn the contact model. Black points represent the five highest local features used to learn the task. The global reference frame of the object, e.g. at the base of the teapot (middle), is not considered in the learning.

In contrast, this paper proposes one-shot learning for aerial manipulation in which contacts are not solely learned from local features but also encode intrinsic task-dependent knowledge without needing handcrafted features, as visible in Figure 1. The underpinning idea is simple. Imagine having to connect a load to a single drone. If the load is a box with uniform mass distribution, probably the best thing we could do is attach the drone in the middle of the top-facing surface, just above the payload’s CoM. In order to find the desired spot, we do not need to have a perfect model of the object or see it in its completeness. A view of its entireness of the top-facing surface would be sufficient for estimating its centre. Nevertheless, counterexamples come to mind where merely teaching the robot to attach the payload above its CoM may not be a robust strategy; a package with overlapping ridges makes the desired contact area irregular and unsuitable for reliable contact. If we have taught the robot to connect over flat surfaces, we desire to maintain this property on novel objects. Hence, our approach weights the contact’s local shape versus its relative location over the visible payload’s surface. The latter is implemented as a probability distribution over the distances between the taught contact regions and sampled patches that describe the general shape of the visible geometry of the payload, e.g., flat surfaces, edges and corners. The main drawback of this approach is that we will need to learn a new contact model for each desired task and contact type. In robot manipulation, a grasp encodes the desired contacts with the object to be grasped, such as a pinch or power grasp. These grasp models generalise well over object categories, but the grasp model has no knowledge of the task. Further (context-dependent) information is needed to decide when a pinch grasp is more suitable than a power grasp. By including the task information in the model, e.g., transporting a load with uniform mass distribution, we lose this decoupling and new models need to be learned.

We evaluate our proposed approach in a set of empirical experiments and compare the results against a baseline method. Our aim is to demonstrate that our approach successfully captures the task from the training sample while a more conventional contact-based approach would fail. First, we have modified the approach in (Kopicki et al., 2016) to cope with a single or multiple UAVs equipped with cable-suspended passive grippers in lieu of a dexterous manipulator. From the same training data, we learned contact models for the baseline and our approach. Then, we use the learned models to generate candidate contact points for four test payloads. The contacts have been evaluated in a simulated transportation task, where the UAVs need to lift the payload and transport it along a desired trajectory, showing that our inferred contacts are more suitable for the given task.

**FIGURE 2:**
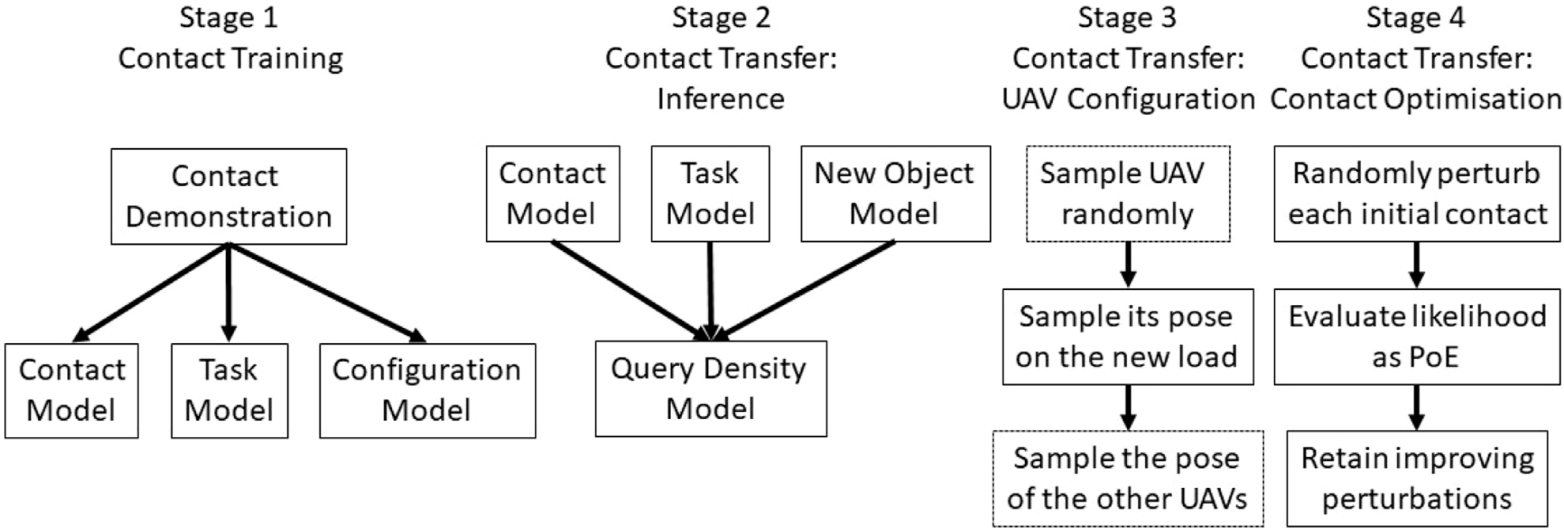
Full pipeline of our method. Stage 1 represents the training of the models. From one single example we extrapolate the three models as probability density functions. Inferring the contact on a new load is decomposed in the three phases: Stage 2 samples similar local contacts on the new object; Stage 3 samples a feasible configuration for the UAV(s); and Stage 4 optimises the contacts and configurations for a better solution, if any. The baseline approach uses the same pipeline but without the task model. For a single UAV Stage 3’s dotted blocks are disregarded.

In summary, our main contribution is the investigation of a contact-based grasp synthesis approach for dynamic tasks. Without physical knowledge of the payload optimal solutions cannot be guaranteed, but our simulated experiments demonstrate that our approach leads to more reliable contacts. The full pipeline of our approach is presented in Figure 2. For clarity’s sake, we identify the same 4 stages as presented in the original work of our modified baseline (Kopicki et al., 2016). While the baseline only learns a contact and configuration models from the training, we extend this formulation with the task model. Further details are available in the caption.

The rest of the paper is structured as follows. In Sec. 2, we introduce the problem formulation in terms of object-centric representation of the contacts and the robot mechanics. Section 3 describes how we learn the models needed for aerial manipulation. Section 4 presents how we infer contacts on novel shapes. The experimental evaluation is presented in Section 5 and our results are discussed in Section 6. We conclude with our final remarks about the strengths and limitations of this work and future research directions.

## 2 Problem formulation

Let us begin from defining our notation. Vectors will be consider column vectors and written in bold letters. Matrix will be written as capital letters. We also denote by $\mathrm{SE}\left(3\right)=R^{3}\times SO\left(3\right)$ the standard Euclidian group in a three-dimensional space, and by $SO\left(3\right)=\left\{R\in R^{3\times 3}|R^{⊤}R=I_{3\times 3},\det\left[R\right]=1\right\}$ the special orthogonal group representing rotations in the three-dimensional space. To describe rigid body transformations or poses, we will use the following format of denoting *v* = (**p**, **q**) ∈ SE (3) where $p\in R^{3}$ is the translational component and **q** ∈ *SO*(3) is the quaternion describing the rotational component. Without losing generality, we abuse of the bold notation for the quaternion since they are implemented as a column vector in $R^{4}$ .

### 2.1 Mechanics

We consider a set of *N*_*q*_ ≥ 1 quadrotors with cable-suspended magnetic grippers. The inertia frame I is defined by the unit vectors **e**_*x*_ = [1,0,0]^*⊤*^, **e**_*y*_ = [0,1,0]^*⊤*^, and $e_{z}={\left[0,0,1\right]}^{⊤}\in R^{3}$ , and the third vector is aligned opposite to the direction of gravity. For each quadrotor, we define a body-fixed frame $B_{n}=\left[b_{x},b_{y},b_{z}\right]$ located at the centre of mass (CoM) of the quadrotor with its third axis aligned upward.

The pose *b*_*n*_ = (**p**_*n*_, **q**_*n*_) ∈ SE (3) describes the location of the CoM of the *n*-th quadrotor, $p_{n}\in R^{3}$ , and its rotation with respect to the inertia frame, **q**_*n*_ ∈ *SO*(3). The mass and the inertia matrix for each quadrotor are denoted as $m_{n}\in R$ and $J_{n}\in R^{3\times 3}$ , respectively. We also denote the control input for the quadrotor as the pair (*f*_*n*_, **M**_*n*_), where $f_{n}\in R$ is the total thrust and $M_{n}\in R^{3}$ the generated moment with respect to its body frame. With respect to the inertia frame, the quadrotor can generate a thrust $fR\left(q\right)e_{z}\in R^{3}$ , where *R*(**q**) ∈ *SO*(3) is the equivalent rotation matrix to the quaternion **q**.

Each quadrotor is equipped with a cable of length *l*_*n*_ and let $\xi_{n}\in S$ be the unit-vector representing the direction of the *n*-th cable pointing outward from the quadrotor’s CoM to the gripper. Let *L*_*n*_ ∈ SE (3) be the pose for the end-effector’s link for the *n*-th quadrotor with respect to the inertia frame. The contact points are defined with respect to the payload’s reference frame, *z* ∈ SE (3), where $m_{z}\in R$ and $J_{z}\in R^{3\times 3}$ represent the mass and the inertia matrix, respectively.

### 2.2 Surface features

**FIGURE 3:**
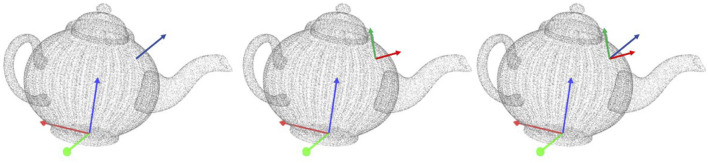
Computation of a surface feature on a teapot. (left) the full point cloud of the teapot with its reference frame. The normal of the sampled point is shown as a blue arrow (middle) the principal curvature directions are drawn for the same point as red and green arrows (right) the associated reference frame for the surface feature with respect to the object’s reference frame.

Surface features encode the geometrical properties of an object and are derived from a 3D point cloud, as shown in Figure 3. We represent a surface feature as a pair $s=\left(v,r\right)\in \mathrm{SE}\left(3\right)\times R^{2}$ , where *v* = (**p**, **q**) ∈ SE (3) represents the pose of the surface feature *s* and $r\in R^{2}$ is a vector of the surface descriptors. All poses denoted by *v* are specified relative to the inertia frame I .

To compute the pose *v* ∈ SE (3), we first estimate the surface normal at point **p** using a PCA-based method (Kanatani, 2005). Surface descriptors correspond to the local principal curvatures around point *p* (Spivak, 1999), which lie on the tangential plane to the object’s surface and perpendicular to the surface normal at *p*. Let $k_{1}\in R^{3}$ be the direction of highest curvature, and $k_{2}\in R^{3}$ the direction of lowest curvature perpendicular to *k*_1_. Let us also define $r={\left[r_{1},r_{2}\right]}^{⊤}\in R^{2}$ as a 2D feature vector representing the curvatures along directions *k*_1_ and *k*_2_, respectively. The surface normal and principal directions form a body-fixed frame for the surface point **p** and enable us to compute the 3D orientation **q** that is associated to the point.

### 2.3 Kernel density estimator

In this work, probability density functions are approximated *via* kernel density estimation (KDE) (Silverman, 1986), which are built around surface features (see Section 2.2). A kernel can be described by its mean point *μ* ^*s*^ = (*μ*_*p*_, *μ*_*q*_, *μ*_*r*_) and bandwidth *σ* ^*s*^ = (*σ*_*p*_, *σ*_*q*_, *σ*_*r*_):

$$K\left(s\;|\;\mu^{s},\sigma^{s}\right)\;=N_{3}\left(p\;|\;\mu_{p},\sigma_{p}\right)\;\Theta \left(q\;|\;\mu_{q},\sigma_{q}\right)\;N_{2}\left(r\;|\;\mu_{r},\sigma_{r}\right)$$ (1)

where *s* = (*v*, **r**) = (**p**, **q**, **r**) is the surface feature being compared against the kernel, $N_{k}$ is an *k*-variate Gaussian distribution, and Θ corresponds to a pair of antipodal von Mises-Fisher distributions forming a distribution similar to that of a Gaussian distribution for *SO*(3) (Fisher, 1953).

Given a set of *N*_*s*_ surface features, the probability density in a region of space is computed as the local density of features in that region, as

$$P\left(p,q,r\right)\equiv P\left(s\right)≃\overset{N_{s}}{\underset{i=1}{\sum }}w_{i}K\left(s|s_{i},\sigma^{s}\right)$$ (2)

where *s*_*i*_ corresponds to the *i*-th surface feature acting as a kernel, *w*_*i*_ corresponds to its weighting with the constraint $\sum_{i=1}^{N_{s}}w_{i}=1$ , and *σ* ^*s*^ is a user-defined bandwidth for the surface features.

## 3 Learning contacts

We learn contacts from a single demonstration. We require a point cloud of the payload and the configuration of the quadrotors and their passive grippers. Partial views of the objects are sufficient for learning reliable models under the assumption that the surface in contact is fully visible. Figure 1 (left) shows the case in which only the top face of a box is visible at training time. Although learning contacts is computational efficient, as demonstrated in (Kopicki et al., 2016), the time computation grows linearly with the number of links in contact, the number of triangles in the mesh representing the links, and the surface points considered. Furthermore, a new model needs to be learned for different drone configurations, contact types or tasks.

### 3.1 Object and task model

The object model describes the composition of a point cloud in terms of its surface features distribution. In the literature, this approach is used to learn the features distribution only nearby the contact area, e.g., (Kopicki et al., 2016; Stüber et al., 2018; Arruda et al., 2019), while in (Howard and Zito, 2021) the CoM of the object to be pushed is also estimated as a distribution from visible surface features. In contrast, we decouple the object model into two densities. The first describes the surface features distribution nearby the contacts, while the second encodes the location of these near-the-contact features with respect to other features in the visible point cloud, e.g., its corners and edges.

At training time, we observe a set of contacts between the quadrotors’ manipulative links and an object point cloud. Before learning a contact model (see Section 3 2), we collect a set of *N*_*O*_ features, $s_{i}=\left(v_{i},r_{i}\right)\in \mathrm{SE}\left(3\right)\times R^{2}$ , within the surface in contact with the manipulative link. This enables us to compute a joint probability distribution as

$$O\left(v,r\right)\equiv P\left(v,r\right)≃\overset{N_{O}}{\underset{i=1}{\sum }}w_{i}K\left(v,r|s_{i},\sigma^{s}\right).$$ (3)

further referred as the *object model* as in (Kopicki et al., 2016), where the weight *w*_*i*_ = 1/*N*_*O*_. Figure 1 shows in red the local area considered for extrapolating the features for two different contact types: a) flat contact and b) curved contact.

**FIGURE 4:**
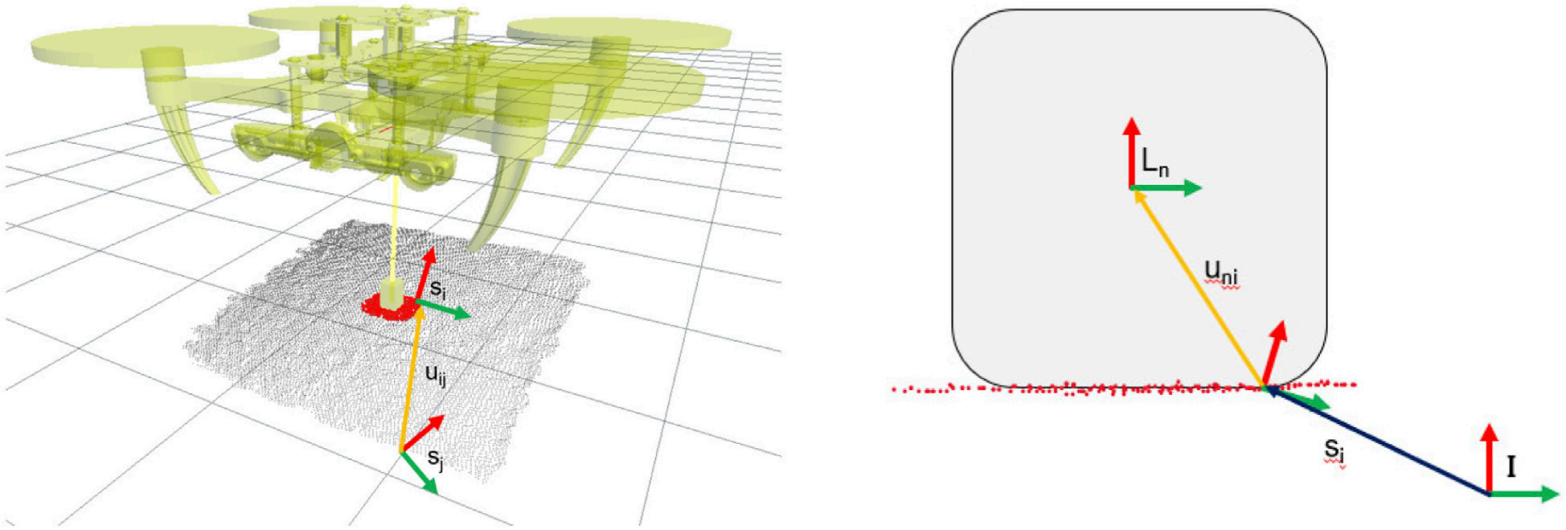
Graphical representation of the task model (left) and the link pose (right) used in the contact model. The task model is constructed as a rigid body transformation *u*_*ij*_ in SE (3) from a task feature *s*_*j*_ to a surface feature *s*_*i*_ in the contact region. The link pose is computed as a rigid body transformation *u*_*ni*_ in SE (3) from a surface feature *s*_*i*_ in the contact region to the reference frame of the robot’s link. All the SE (3) poses *s*_*j*_, *s*_*i*_, *L*_*n*_ are computed with respect to the inertia frame I .

From the same point cloud, we collect a second set of *N*_*T*_ features by uniformly sampling the visible point cloud to compute a second joint probability as

$$T\left(v,r\right)\equiv P\left(v,r\right)≃\overset{N_{T}}{\underset{j=1}{\sum }}w_{j}K\left(v,r|s_{j},\sigma^{t}\right).$$ (4)

further referred as the *task model*, where $s_{j}=\left(v_{j},r_{j}\right)\in \mathrm{SE}\left(3\right)\times R^{2}$ , *σ* ^*t*^ is the bandwidth and *w*_*j*_ = *r*_1*j*_/*r* is the weight associated with the feature represented by the *j*-th kernel and it is proportional to its highest curvature normalised with respect to the maximum curvature value available between the *T*_*s*_ sampled points, *r*. This enables us to give more importance to salient features such as corners and edges. Figure 4 shows a visual representation of the task model.

### 3.2 Contact models

The contact model describes the relation between the robot’s end-effector and the object surface. It is trained in a one-shot fashion from a demonstration. A teacher presents to the robot the desired contact, either in simulation or in reality, then the local surface in contact with the robot’s link is sampled, and a probability density function is learned.

We denote by *u*_*ni*_ = (**p**_*ni*_, **q**_*ni*_) ∈ SE (3) the pose *L*_*n*_ of the *n*-th link relative to the pose *v*_*i*_ of the *i*-th surface feature from (3). We compute this as

$$u_{ni}=v_{i}^{-1}◦L_{n}$$ (5)

where ◦ denotes the pose composition operator, and $v_{i}^{-1}=\left(-q_{i}^{-1}p_{i},q_{i}^{-1}\right)$ is the inverse of pose *v*_*i*_.

The contact density *M*_*n*_ (*u*, **r**) closely resembles the surface feature kernel function in (1), and is defined as follows:

$$M_{n}\left(u,r\right)≃\overset{N_{c}}{\underset{i=1}{\sum }}w_{ni}N_{3}\left(p\;|\;p_{ni},\sigma_{p}^{c}\right)\;\Theta \left(q\;|\;q_{ni},\sigma_{q}^{c}\right)\;N_{2}\left(r\;|\;r_{ni},\sigma_{r}^{c}\right)$$ (6)

where *N*_*c*_ ≤ *N*_*O*_ is a user defined parameter to allow downsampling to save computational time and *w*_*ni*_ corresponds to the kernel’s weighting such that $\sum_{i=1}^{N_{c}}w_{ni}=1$ .

### 3.3 Quadrotor configuration model

The configuration model encodes the poses of the quadrotors and their grippers as demonstrated during training. For a single drone, the configuration model merely describes the kinematic relation between the quadrotor and its gripper. However, when *n* > 1, this model enables us to reduce the configuration space when transferring the contacts to another surface by focusing only on those configurations that resemble the one in the training example, e.g., a triangular formation. The configuration model is not capable of choosing the correct number of UAVs. It reinforces the learned formation onto a novel payload. The SA optimisation can locally optimise the pose of the single UAV in order to generate a higher-scoring contact while simultaneously avoiding collisions or kinematically impossible configurations. The room for the local improvements is bounded by a user-provided standard deviation on the expected placement of the gripper for the desired formation. This enables us to cope with different sized and shaped payloads.

The poses of the *N*_*b*_ ≥ 1 quadrotors are represented as *h*_*n*_ = (*b*_*n*_, *L*_*n*_) with *b*_*n*_ = (*p*_*n*_, *q*_*n*_) ∈ SE (3) the pose of the *n*-th drone and $L_{n}=\left(p_{L_{n}},q_{L_{n}}\right)\in \mathrm{SE}\left(3\right)$ the pose of its manipulative link. We approximate the configuration density as

$$H\left(h\right)≃\overset{N_{b}}{\underset{i=1}{\sum }}w_{i}N_{3}\left(p_{L}\;|\;p_{L_{i}},\sigma_{p}\right)\;\Theta \left(q_{L}\;|\;q_{L_{i}},\sigma_{q}^{c}\right)\;$$ (7)

where $w_{i}=e^{-\alpha \|b-b_{i}{\|}^{2}}$ depends on the similarity between the drone’s pose, *b*, and the kernel’s one, *b*_*i*_, while *L* = (**p**_*L*_, **q**_*L*_) is the link pose compared against the *N*_*b*_ link poses from the kernels, $L_{i}=\left(p_{L_{i}},q_{L_{i}}\right)$ .

## 4 Transferring to novel surfaces

Once the models are learned, we can transfer the contacts on novel payloads. The aim is to find local features on the new object that are similar to those presented in training and to place the robot’s manipulative links accordingly. For evaluating the approach, we learn several contact models for different tasks and contact types–we call them *testing conditions* as introduced in Section 5. However, when presented with a new point cloud, we manually select the appropriate contact model for each testing condition. Automatic model selection can be formulated as an optimisation problem (Pozzi et al., 2022) or a learning one (Kober et al., 2013), but we kept it as out of scope for this work.

### 4.1 Query density

**FIGURE 5:**
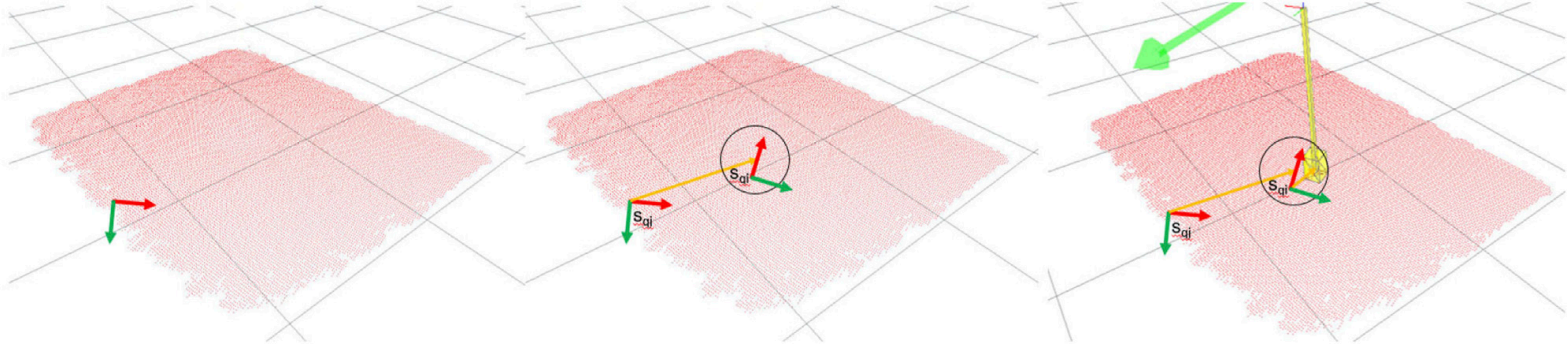
Graphical representation of inferring a candidate link pose for a novel surface. First, from the task model we sample a feature *s*_*qj*_ on the novel point cloud. We then apply the transformation *u*_*ij*_ observed when learning the model to identify a feasible contact region. From that region (circled area in the middle image), we sample a surface feature from the object model *O* (*v*, **r**). Finally we apply the transformation *u*_*ni*_ observed at training time to place the link *L*_*n*_.

The query density represents the distribution of link poses over a novel point cloud. By searching for local similarities in the query point cloud’s surface features, we can estimate the relative link pose *u*, which transfers the learned contact onto the new surface. This process is designed to be transferable such that a model trained upon a single object can be applied to a variety of previously unseen objects. Figure 5 shows a graphical representation of the method: starting from a sampled task feature, we identify a potential area on the visible surface where to seek for a good contact.

We define the query density as *Q* (*L*_*n*_, *u*, *v*, **r**) where *v* ∈ SE (3) denotes a point on the object’s surface expressed in the inertial frame, $r\in R^{2}$ is the surface curvature of such a point, *u* ∈ SE (3) denotes the pose of the link relative to a local frame on the object, and *L*_*n*_ is the pose of the *n*-th link with respect to the inertia frame.

For each link, its pose distribution over a new point cloud is given by marginalising *Q* (*L*_*n*_, *u*, *v*, **r**) with respect to *u*, *v*, and **r**. Since *L*_*n*_ = *v*◦*u* is described in terms of *v* and *u*, and by assuming that the density in the inertia frame of the surface point, *v*, and the distribution of link poses relative to a surface point, *u*, are conditionally independent given the curvature on a point, **r**, we factorise the query density as follows

$$\begin{array}{ll} Q\left(L_{n}\right)= & ∭P\left(L_{n},u,v,r\right)dudvdr∭P\left(L_{n}|u,v\right)P\left(u,r,v\right)dudvdr∭P\left(L_{n}|u,v\right)P\left(u|r\right)P\left(v|r\right)P\left(r\right)dudvdr \end{array}$$ (8)

where, by following (Kopicki et al., 2016), we implement *p* (*L*_*n*_|*u*, *v*) as a Dirac function, and *p* (*u*|**r**) as *M*_*n*_ (*u*|**r**), the conditional probability that the *n*-th link will be placed at pose *u* with respect to the surface feature, given that this surface feature has curvature **r**. *p* (*v*|**r**) is implemented as *O* (*v*|**r**), the probability of the observed curvature **r**. Finally, *p*(*r*) = *M*_*n*_(**r**)*O*(**r**) is chosen to reinforce that the contact model *M* and the object model *O* are observing the same surface feature. *M*_*n*_(**r**) is the distribution of features in the contact model, and *O*(**r**) is the distribution of feature **r** in the new point cloud.

We extend this formulation with another random variable *u*_*ij*_ = (**v**_*ij*_, **q**_*ij*_) to encode the desired pose of the selected *i*-th surface feature of the contact model relative to the task model’s features defined in (4). Thus, $u_{ij}=v_{i}^{-1}◦v_{j}$ , where *s*_*j*_ = (*v*_*j*_, *q*_*j*_) belong to the task model. Then we approximate the query density by sampling *N*_*Q*_ kernels centred on weighted link poses from (7), so that

$$Q\left(L_{n}\right)≃\overset{N_{q}}{\underset{k=1}{\sum }}w_{nk}N_{3}\left(p\;|\;p_{k},\sigma_{p}\right)\;\Theta \left(q\;|\;q_{k},\sigma_{q}\right)$$ (9)

where (**p**_*k*_, **q**_*k*_) ∈ SE (3) describes the *k*-th kernel. The variable

$$w_{nk}=\frac{1}{Z}\overset{N_{i}}{\underset{i=1}{\sum }}P\left(L_{n}|u_{ni},v_{i}\right)M\left(u_{ni}|r_{i}\right)M\left(r_{i}\right)O\left(v_{i}|r_{i}\right)O\left(r_{i}\right)\left(\overset{N_{j}}{\underset{j=1}{\sum }}P\left(u_{ij}|r_{i},r_{j}\right)T\left(v_{j}|r_{j}\right)T\left(r_{j}\right)\right)$$

weights the query density according to the contact model and the task model. To compute the weighting, we randomly sample from the new point cloud *N*_*i*_ surface features from the estimated contact surface and *N*_*j*_ features according to the task model so that *s*_*j*_ ∼ *T* (*v*_*j*_|**r**_*j*_) is the observed feature **r**_*j*_ on the new point cloud. Again, we implement *p* (*u*_*ij*_|**r**_*i*_, **r**_*j*_) as a Dirac function and *T* (*v*_*j*_|**r**_*j*_)*T* (**r**_*j*_) represents the probability of the observed curvature on the new point cloud. The value *Z* is a normaliser, and the value *N*_*i*_ and *N*_*j*_ are maintained constant across all the experiments.

### 4.2 Contact optimisation and selection

Let us denote by $\hat{h}={\left\{\left(b_{i},L_{i}\right)\right\}}_{i}^{n}$ the poses of the quadrotors and their gripper in SE (3). The objective of the contact optimisation is to generate candidate contacts by maximising the product of the query densities and the quadrotors configuration density, as follows:

$${arg\;max}_{\hat{b}}J\left(\hat{h}\right)={arg\;max}_{\left(b_{i},L_{i}\right)}\underset{\left(b_{i},L_{i}\right)\in \hat{b}}{\prod }H\left(h_{i}\right)Q\left(L_{i}\right)$$ (10)

where J is the likelihood of the candidate contact on the new point cloud. We optimise the likelihood using simulated annealing (SA) (Kirkpatrick et al., 1983).

## 5 Results

In this section we present our empirical results aiming to demonstrate that task-dependent features can be encoded in the contact models without the need of heuristics or prior knowledge. The proposed approach has been evaluated in a set of simulated experiments on several conditions. For a single UAV, we learn two types of contacts: (i) a contact placed at the centre of the visible upper surface of a box, and (ii) a contact on a curved surface. For multiple drones, we learn a triangular formation for three UAVs for a transportation task.

In all conditions, we learn each contact model in one single demonstration. Each training object was loaded in the simulation as full or partial point cloud, as presented in Figure 1. We do not retrieve global information about the object, such as its CoM. The UAVs were manually placed to generate the desired contacts with their passive gripper over the visible point cloud. For all conditions, we sampled *N*_*O*_ = 500 local features to represent the contacts in both our approach and baseline method and *N*_*T*_ = 50 for the task model (present only in our approach). Once a contact model was learned, it was stored and labelled for future reference. Only kinematic and geometrical information was used to learn the models; forces and dynamics are not needed for learning.

**FIGURE 6:**
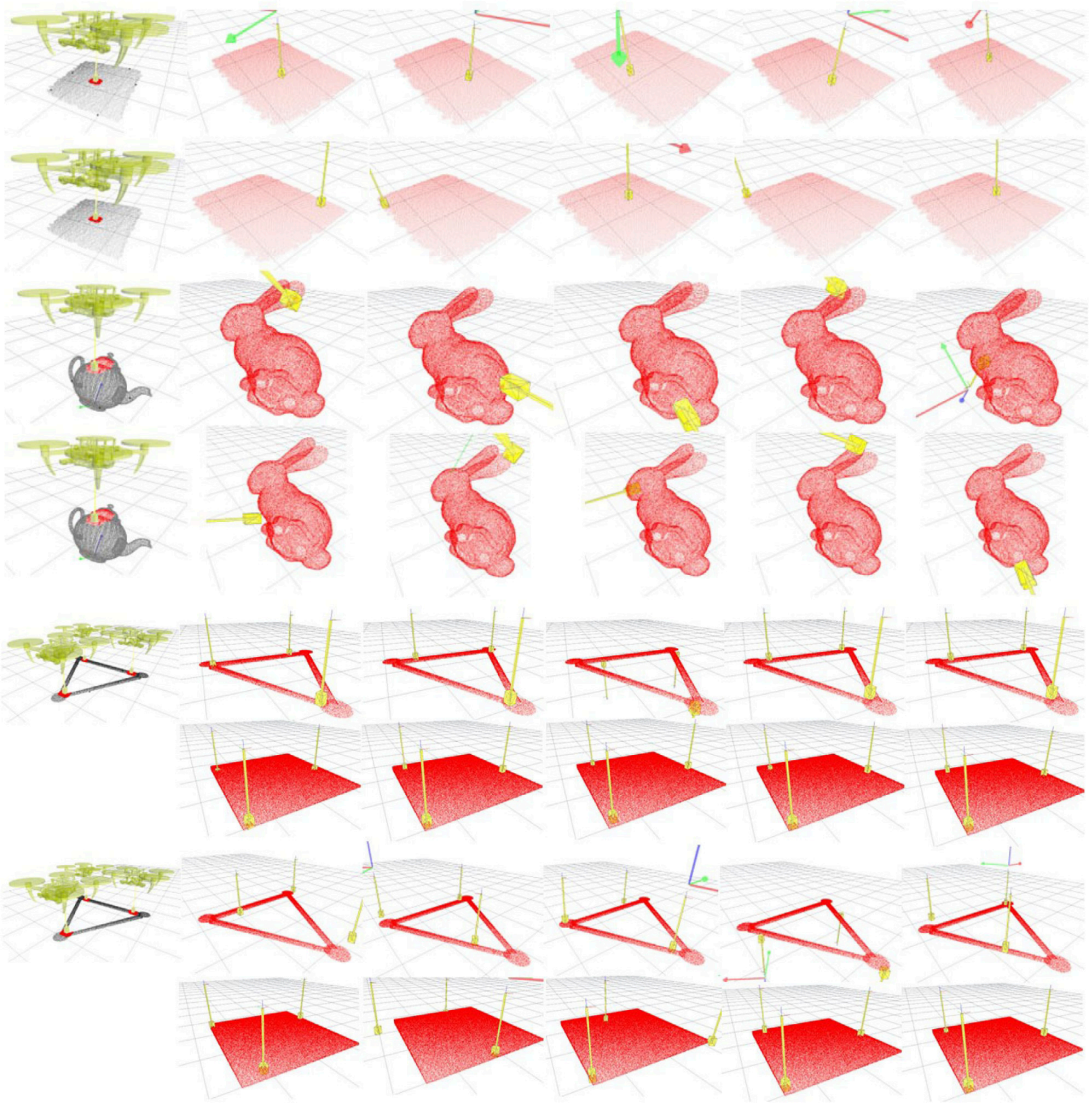
Contact inference on a new shape for aerial manipulation. The first column shows the training examples used for learning the contact models. The first, third, fifth and sixth rows show the transferring of the contacts for our approach. The other rows show the baseline method (Kopicki et al., 2016). The first column shows the UAVs configuration, the surface used for learning and the contact features. Red points are the local features of the contacts; black points are the task features. The other columns demonstrate the link pose computed on a new surface. The associated drone’s pose is shown with a frame using the conventional RGB colour map for x-, y-, and z-axes, respectively.

Once the contact models are learned, a new full or partial point cloud is presented to the system. In Figure 6 we show the point clouds belonging to four objects used for testing: a) 68cm x 61 cm x 28cm FedEx box (top two rows); b) the Stanford bunny (third and fourth rows); the hollowed triangular shape used to learn the three drones’ contact models (fifth and seventh rows); and a 68cm x 68 cm x 28cm FedEx box (sixth and eighth rows). We then compare the best five solutions generated for each condition with those computed by an adapted version of the grasping algorithm presented in (Kopicki et al., 2016). Since (Kopicki et al., 2016) considers a robot manipulator equipped with a hand, we modify it to consider the quadrotor configuration model. Therefore, the main difference between the two approaches remains the task model.

### 5.1 Contact inference: a qualitative analysis

Our first experiment aims to compare how our model infers contacts on a novel surface against the baseline method. Figure 6 presents the results. For the purpose of qualitative analysis, we can identify the following four conditions: C1) single UAV with a flat contact (row 1 and 2 in Figure 6), C2) single UAV with a curved contact (row 3 and 4), C3) three UAVs with no generalisation (row 5 and 7), and C4) three UAVs with generalisation to a novel shape (row 6 and 8). The rows in Figure 6 for each condition are directly comparable, showing our method’s solutions against the baseline’s ones, respectively.

In condition C1, we learn a flat surface contact on a squared box and test on a rectangular-shaped box. The taught contact lays in the middle of the upper face of the box, ideal for transportation of payloads with uniform mass distribution. Under the assumption that no more information is available on the physical properties of the load but that of uniform mass distribution, it is safe to assume that even a human operator would attempt to generate contacts in the middle of the upper face of the rectangular box. We encode this strategy in the training example. Nonetheless, a more conventional contact-based algorithm such as the baseline is not capable of extrapolating this subtlety. The load presents a large number of possible solutions in which the gripper is in contact with a flat surface. All these candidates are evaluated according to local surface features extrapolated from the point cloud, as shown in Eq. 8, and feature-rich areas would uncontroversially score higher values. Hence, flat surfaces nearby corners or edges look more promising for such methods. In contrast, our task model forces the contacts away from such areas, giving the method a sort of global, task-dependent understanding of the payload’s geometry. Yet, thanks to the soft probabilistic representation of the task model, our approach can deal with the different shape of the test payload, adapting the learned contact on a squared surface onto a rectangular one. The first and second rows in Figure 6 show that the five best candidates for our method are clustered towards the middle of the rectangular face, while the baseline’s solutions span the entire surface.

Condition C2 presents a less intuitive example in which a clear optimal solution may not be identifiable on a bunny-shaped payload. The contact model trained in C1 would not be helpful in this case–and it should not be expected to–since it would seek flat contacts on a mostly curved surface. We, therefore, demonstrate to the system how to generate contacts on a curved surface using a teapot as a payload. The contacts we demonstrated take advantage of the teapot’s symmetry and lay in the centre to have its handle and beak on opposite sides. Because of the irregular shape of its lid, we placed the contacts on its body’s smoother surface just below the lid’s edge. Without the need to encode this strategy into the model explicitly, our approach seems to capture the essence. The solutions presented in the third row of Figure 6 are clustered in the mid-section of the bunny taking advantage of its symmetry but avoiding the more irregular areas on its tail and snout. In contrast, the baseline solutions shown in the fourth row only maximise the similarity in the local surfaces between the taught contact points and the new point cloud. In fact, its best solution is placed on the edge of the bunny’s chest; a large, smooth surface area with a similar curvature to the one on the teapot. Nevertheless, as demonstrated in the transportation task, contacts in this area lead to high swings when the payload is lifted. Its next three solutions are also far from the bunny’s mid-section: two on the edge of the ears and one on the face, making the payload difficult to be controlled along the desired trajectory.

In conditions C3 and C4, we test the ability of the algorithms to deal with multiple contact points simultaneously. We demonstrate a tripodal grasp with three UAVs in a triangular formation on a hollowed triangle shape. We represent the robot configurations as a virtual robot hand’s three kinematic chains or *fingers*. Similarly to any other contact-based grasping algorithm in the literature, different finger configurations and contacts characterise each grasp type; thus, the tripodal contact model must be used with three UAVs and cannot scale up or down. Another model would need to be learned for a different number of drones or configurations (e.g. three drones in a straight line). However, each UAV’s pose can be adjusted to adapt the grasp to a different payload thanks to the soft probability representation of the configuration model presented in Section 3.3.

Condition C3 does not attempt to generalise to a novel shape. Three contacts must be found on the triangle’s surface, and the query distribution treats that as an optimisation problem. It randomly selects one UAV and finds a suitable contact, with the same procedure used for the single UAV case. It then places the other drones according to the formation presented in the training data. The optimisation allows local modifications of all three UAV configurations to maximise the sum of each contact’s score. Similarly to the single UAV case, many local patches on the triangle provide flat contacts to initialise the procedure, but not all lead to a reliable tripodal grasp. This yields to local minima observable in the baseline, which often cannot find a three-contact placement on the challenging surface. In contrast, our task model helps the optimisation procedure identify better initial guesses and yields more reliable solutions. We extrapolate the same conclusion from the last condition in which the tripodal grasp is generalised on a squared flat surface. In this condition, it is even more observable how the baseline chooses its initial guess nearby feature-rich areas of the test objects, i.e. corners and edges, whilst our approach is not biased by it.

### 5.2 Transportation task: a quantitative analysis

**FIGURE 7:**
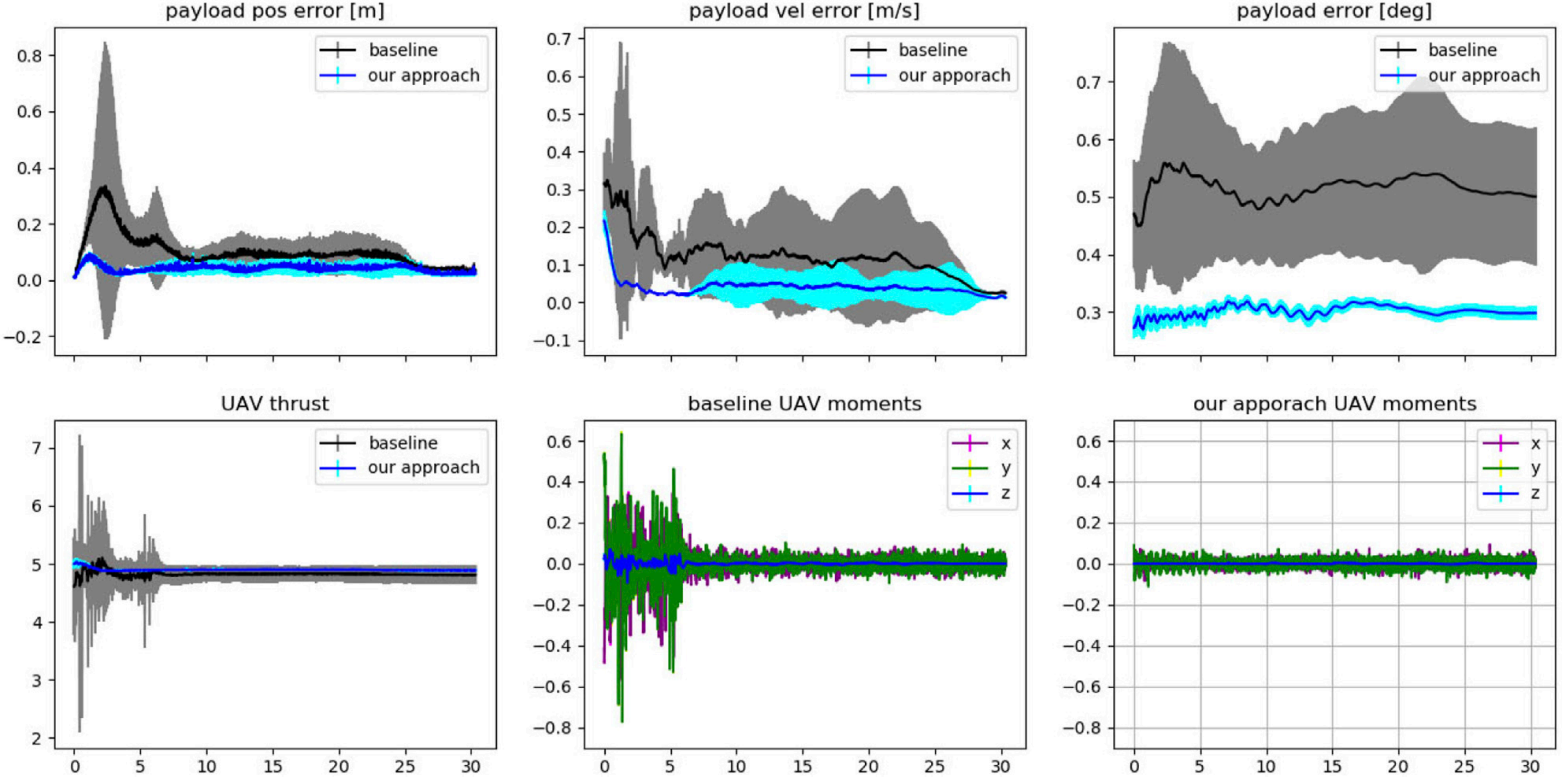
The results summarise the transportation experiments. Each solution from Figure 6 is used for ten trials in a simulated environment in which the single or multiple UAVs lift the payload, transport it along a circular trajectory twice, and then hover the payload above ground for at least 5 seconds. The *x*-axis represents the time in seconds for all plots. The top row shows the averaged position and velocity error w.r.t. the desired payload’s trajectory (baseline and proposed method separately), and both averaged rotational errors in the last plot. The bottom row shows the UAV’s thrust (together) and moments (separately). For the three UAVs condition, the values in the bottom row have been averaged for each UAV.

Each solution from Figure 6 is also evaluated in a simulated environment for a transportation task. The simulator is initialised with the computed optimised contacts. The UAVs will need to transport the payload along a pre-planned circular trajectory with a 1 m radius performed for 12 seconds. The trajectory is computed such that the UAVs need to lift the payload to the starting position of the trajectory, perform the circle twice and then hover above the ground, maintaining the payload in the same position as the last waypoint of the trajectory. The simulation uses the PCMPC controller as in (Li et al., 2021b), also adapted by us to control a single UAV with a payload that is not a point mass object. Additional, Gaussian noise with 1cm standard deviation was applied to the perceived position of the payload, and each transportation task for each contact was repeated ten times. The results were averaged across each object and trial and presented in Figure 7.

The solutions for the hollowed triangular shape are from the complete point cloud of the object, for which solution 3 for our approach and solution 4 for the baseline suggested an upside-down contact. We discarded these solutions from the transportation task since we could not achieve the required kinematic of the contact. All the other solutions from the baseline methods that do not provide contacts for each UAV have been modified to connect the gripper to the closest point on the payload. Considering the kinematically infeasible solutions for the three drones condition, we had a total of 147 runs (3 contact models x 5 solutions x 10 trials −3 infeasible solutions) for the proposed model and 147 for the baseline. In all conditions, we use a set of fixed parameters as shown in Table 1.

**TABLE 1:** Experimental parameters. All of the parameters are kept constant over the experiments. We use diagonal inertia matrices for both the UAVs and payloads. The gains values are for the *x* −, *y* −and *z* −components, respectively. UAV and gains parameters are taken from (Li et al., 2021b).

Contacts		UAV		Payload		Gains		
Learning		*m*_*n*_	250 g	*m*_*z*_	250 g	pos	Controller	Cable
*N*_*O*_	500	$J_{n}^{xx}$	0.0006	$J_{z}^{xx}$	0.006	6, 6, 10	1.45, 1.45, 3
*N*_*T*_	50	$J_{n}^{yy}$	0.0005	$J_{z}^{yy}$	0.0059	vel	3, 3, 6	3.7, 3.2, 2.5
*N*_*C*_	500	$J_{n}^{zz}$	0.001	$J_{z}^{zz}$	0.107	Rot	0.25, 0.25, 0.08	0.5, 0.5, 0.02
Inferring		*l*_*n*_	0.5 m			ang	0.025, 0.025, 0.005	0.16, 0.16, 0.04
*N*_*i*_	500	# prop	4			*ξ*	16, 16, 16	
*N*_*j*_	5	max RPM	16400			*ω*	6.7, 6.7, 3.1	
*N*_*Q*_	1000	min RPM	5500					

In Figure 7 we observe the averaged difference in transporting the payloads given the choice of the contacts. Our approach outperforms the baseline in terms of accuracy in following the pre-planned payload trajectory and by minimising the effort computed by the UAVs as thrust and moments. Interestingly, in all conditions, our approach enables the UAV to control the orientation of the payload in a more agile way. This is due to the relative position of the contact points with respect to the payload’s shape, which minimises oscillations during the transportation. This is clearly visible by the lower standard deviation from the desired position and orientation of the payload associated with our approach. Furthermore, the velocity profile of the payload and the output to the controller (thrust and moments) show that our contact points simplify the work of the controller in following the desired path.

The next section will present our final remarks.

## 6 Conclusion

This paper proposed a framework for learning task-dependent contacts for aerial manipulation. Our models are learned in a one-shot fashion and do not require a complete CAD model of the payload or dynamic modelling. We build on a contact-based formulation. Typically, such methods rely only on local information, and task-dependent features must be handcrafted. In contrast, thanks to the task model presented in Section 3.1, we capture meta-information regarding the task by merely looking at the geometrical features of the point cloud, without the need for user-specific insight about the task.

Although performed in simulation, the empirical evaluation shows the potential of the proposed idea. Without physical and dynamic information about the system, a full convergence to an optimal solution is impossible to achieve. Nonetheless, such information is rarely available in reality, and this work is the first step toward autonomous contact selection for aerial manipulation.

## 7 Strengths, limitations and future work

The presented work extends a (static) contact-based formulation of grasp synthesis to a dynamic task. While previous efforts have focused on integrating an approximation of such dynamics in the models, we investigate how much knowledge about the task we can capture by disregarding the dynamics altogether.

The probabilistic formulation could be considered as a soft simultaneous optimisation of multiple experts. Each expert learns some of the characteristics of the contacts within the feature space, e.g. the task model, and weighs their opinion in the choice of the candidate contact at query time. Any soft simultaneous optimisation of multiple criteria would work in principle. However, the probabilistic representation allows us to draw contact points from a continuous representation of the payload’s surface. Nevertheless, the experts only weight geometrical properties, which do not play a key role in defining the dynamic behaviour of the payload. We assume that the demonstration encodes the contact type (e.g., flat contact), the task (e.g., contact above the payload’s CoM for a transportation task) and the visible surface of the object provides enough salient features to learn a good task model. Although there is no need for handcrafting task features into the model, the training data choice becomes crucial.

Furthermore, our approach assumes that the contact types and tasks are conditionally independent in our model. However, this is generally not true, as seen in the transportation of a FedEx box and the teapot examples. Applying the model learned on the FedEx over the Stanford bunny will lead to a poor choice of contacts since flat surfaces are quite limited in the chosen payload. Therefore, generalisation across contact types and tasks becomes harder to achieve. This also limits the use of the same contact model for a different task (e.g., flat contact next to the edge for dragging the payload) which will require learning a new model.

With many models that need to be learned, we also need to solve the model selection problem when facing a novel payload. Our empirical evaluation disregards this problem by manually providing the correct model at testing time. Reinforcement learning techniques can be used for model selection, but the task would need to be encoded a more informative way for this technique to converge to a good reward function.

The choice of the principal curvatures as surface features is also limiting. It is a baseline for determining the contact points and the general shape of the payload. However, other features may provide better information about the intrinsic dynamic properties of the payload or the encoding task, such as surface roughness or intensity features–or more likely a combination of multiple features.

In order to achieve a fully autonomous aerial manipulation, all these problems will need to be addressed. This work pioneers the problem of grasp synthesis for aerial transportation, but with the increasing interest in aerial manipulation and the technological maturity that we are witnessing, we would suggest that it will become central to many applications. In future work, we will focus on evaluating this framework on a real platform with payloads with different dynamical properties, e.g., mass distribution. Since our approach is a generative model, multiple candidate contacts will be provided for each payload. Primitive dynamic motions could be used to evaluate the selected contacts according to the observed behaviour of the payload.

## Data Availability

The original contributions presented in the study are included in the article/Supplementary Material, further inquiries can be directed to the corresponding author.
